# Aging and vaccines: impact of immunosenescence and inflammaging in vaccine response

**DOI:** 10.3389/fragi.2026.1797166

**Published:** 2026-07-03

**Authors:** Jalnar Albaloshi, Alhussain Alnakhli, Lamin B. Cham

**Affiliations:** Department of Microbiology, Immunology and Infectious Diseases, College of Medicine and Health Sciences, Arabian Gulf University, Manama, Manama, Bahrain

**Keywords:** aging, immunity, immunosenescence, inflammaging, vaccine

## Abstract

Historically, effective vaccination has saved millions of lives and made an enormous contribution to addressing global public health threats. However, aging is an inevitable factor that negatively affects vaccine effectiveness and efficacy. With age, humans develop dysregulation of innate immune pathways, increased levels of proinflammatory markers, thymic involution, declines in T- and B-cell numbers and function, dysregulated metabolism, and epigenetic alterations. Immunosenescence, the gradual deterioration of host immunity with age, plays a critical role in susceptibility to infection, disease progression, and vaccine efficacy. Immunosenescence is often accompanied by inflammaging, an age-related increase in proinflammatory cytokine and chemokine levels. This multifactorial and dynamic process can lead to poor vaccine responses, potentially resulting in higher morbidity and mortality in older adults. Therefore, research focused on understanding the impact of immunosenescence and inflammaging on vaccine response will undoubtedly help optimize vaccine delivery among elderly individuals. In this review, we summarize the most recent knowledge on the multifactorial effects of age-induced immunosenescence and inflammaging and their role in vaccine efficacy against respiratory tract infections. The review further discusses strategic approaches such as IL-7 therapy, senolytic therapy, increasing telomerase activity, use of mTOR inhibitors, B cell therapy, development of broader vaccines and new adjuvants, and positive lifestyle changes, to enhance vaccine immunogenicity and efficacy in older adults. This could provide theoretical insights to improve vaccine effectiveness in the elderly and help address major global health issues.

## Introduction

**FIGURE 1:**
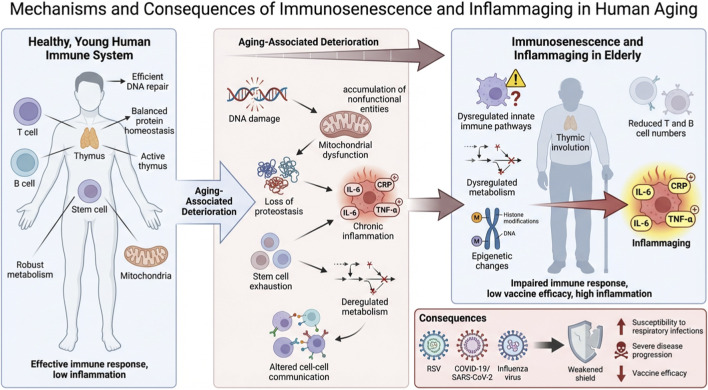
Mechanism and consequences of immunosenescence and inflammaging in human aging: Diagram showing a simplified overview of the age-related decline in cellular, molecular, physiological, enzymatic, hormonal, and metabolic functions, leading to loss of cellular function (Immunosenescence) due to DNA damage, mitochondrial dysfunction, protein imbalance, stem cell exhaustion, inflammation (inflammaging), dysregulated metabolism, and altered intercellular communication.

As humans age, this brings a variety of sequential deterioration across a spectrum of functions at multiple levels, such as cellular, molecular, physiological, enzymatic, hormonal, and metabolic levels (Chen L. et al., 2024; Weiskopf et al., 2009; Xu et al., 2020). Aging is a phenomenon that can lead to a gradual loss of cellular function resulting from DNA damage, mitochondrial dysfunction, loss of protein balance, stem cell exhaustion, inflammation, deregulated metabolism, and altered intercellular communication (Childs et al., 2015; Guo et al., 2022). Aging is associated with the accumulation of harmful changes and nonfunctional molecular entities, thereby affecting cellular and tissue functions (Liu et al., 2023). These age-related changes have important practical implications, including increased risk of cancer, infections, poor vaccine response, cardiovascular diseases, diabetes, and neurological and immunopathological diseases. (Xu et al., 2020; Ikeda and Togashi, 2022). The human immune system is a complex network of cells and proteins that protects against infection. With age, the host immune system gradually declines, increasing the risk of infection and impairing vaccine response (Goyani et al., 2024; Weyand and Goronzy, 2016). Immunosenescence, derived from “immuno-” (referring to the immune system) and “senescence” (the biological process of aging and decline), is an age-related deterioration of the immune system, affecting both its structure and function. Immunosenescence is an age-associated immune dysfunction characterized by dysregulation of innate immune pathways, thymic involution, declines in T- and B-cell numbers and function, reduced repertoire diversity, dysregulated metabolism, and epigenetic alterations (Chen L. et al., 2024; Hou et al., 2024; Goldberg et al., 2020; Pereira et al., 2023). On the other hand, cellular senescence is a multifaceted process that permanently arrests cell proliferation while maintaining metabolic activity. This phenomenon is often triggered by DNA damage, dysfunctional telomeres, specific oncogene expression, disruptions to chromatin organization, and intense mitogenic signals (Regulski, 2017; Ajoolabady et al., 2025; Liu et al., 2025). Quite often, there is confusion between immunosenescence, cellular senescence, and a general immune dysfunction, due to their overlapping roles in aging and the fact that they often drive one another. These phenomena are often mistaken for one another and inappropriately used. In summary, immunosenescence refers to the aging-related decline and deterioration of the entire immune system. Cellular senescence is the permanent halt of cell division induced by stress, while a general immune dysfunction or impairment denotes a broader failure of immune responses that can result from both immunosenescence and cellular senescence (Lee K. A. et al., 2022). Moreover, immunosenescence is often accompanied by inflammaging, a phenomenon characterized by increased levels of proinflammatory markers such as IL-6, C-reactive protein (CRP), and TNF-α (Pinti et al., 2016; Ferrucci and Fabbri, 2018). Given the multidimensional complexity of aging, immunosenescence and inflammaging play a crucial role in driving immunopathology and poor vaccine responses in older adults (Chen L. et al., 2024; Hou et al., 2024). Here, we aim to explore the underlying mechanisms of immunosenescence and inflammaging in vaccine-induced immunity, with a specific focus on vaccines against respiratory syncytial virus (RSV), influenza, pneumococcus, and COVID-19. This review further elucidates the relationship among immunosenescence, poor vaccine response, and increased hospitalization rates due to respiratory tract infections. Figure 1 summarizes the multifactorial effect of aging.

## Hematopoietic aging

Hematopoietic stem cells (HSCs) are multipotent cells that can develop into all types of cells, including immune and non-immune cells (Lee and Hong, 2020). The aging of the hematopoietic system plays a key role in overall aging, affecting immune function, tissue repair, and age-related diseases. Hematopoietic aging involves a decline in HSC function and changes in the bone marrow niche. This is often characterized by myeloid-biased differentiation, decreased lymphopoiesis, reduced regenerative capacity, and a higher frequency of clonal haematopoiesis (Zhang et al., 2020). These age-related changes result from a complex interplay of intrinsic HSC alterations, such as DNA damage, genetic mutations, epigenetic reprogramming, and metabolic dysfunction, and extrinsic factors, such as niche aging and systemic inflammation (Miyawaki et al., 2026; Ye et al., 2024; Fujino et al., 2022). Gene expression profiling showed that aged immunophenotypic human HSCs transcriptionally upregulate genes related to the cell cycle, myeloid lineage specification, and myeloid malignancies (Adelman et al., 2019; Pang et al., 2011). Physiologically, aged mice showed that the increase in myeloid cells with age is due to a greater number of HSC clones contributing to myelopoiesis, rather than to increased myeloid production by individual clones. This refines the understanding of “myeloid bias,” shifting it from an intrinsic lineage skew toward a clonal compensation mechanism. In older adults, peripheral blood frequently exhibits a relative predominance of myeloid cells and a decrease in lymphocyte counts. These age-related changes in the frequency, developmental potential, and gene expression profile of human HSCs are similar to those observed in mouse HSCs. Studies assessing the immunophenotypic profiles of HSC progenitors from healthy young and elderly bone marrow samples found that aged-HSCs are more frequent, less quiescent, and more likely to differentiate into myeloid cells compared to young HSCs (Wouters et al., 2021). An integrative meta-analysis of murine HSC transcriptomes identified an aging signature characterized by an abundance of membrane molecules such as P-selectin and a bias toward upregulation of myeloid genes. This pattern suggests a transcriptionally active state in aged HSCs, evidenced by heightened RNA polymerase II activity and increased chromatin accessibility (White et al., 2026). Transcriptomic analyses in both mice and humans show a decline in lymphoid gene programs with age and a rise in myeloid output, indicating a functional shift. Age-associated somatic mutations (e.g., *DNMT3A*, *TET2*, *ASXL1*) give a proliferative advantage to certain HSC clones (White et al., 2026; Flohr Svendsen et al., 2021). Single-cell analyses of murine HSCs reveal that aging is primarily linked to issues in cell cycle regulation, including changes in G1 phase dynamics and a decline in proliferation-driven function, which in turn affects lineage output. Recent studies of human HSCs show a gradual decline in their ability to respond to mitogenic signals with age. This decline is marked by slower G1 phase progression and decreased AKT signalling, clearly indicating dysregulation of cell cycle control. Another key factor in intrinsic HSC aging is the accumulation of DNA damage from replication stress, oxidative injury, and reduced repair capacity (Montazersaheb et al., 2022a). In mice, aged or DNA repair-deficient HSCs accumulate damage and decline in function, with a p53-dependent differentiation checkpoint restricting self-renewal following genotoxic stress. DNA damage accumulates with age in CD34^+^ HSCs from older individuals. In addition, aging causes widespread epigenetic reprogramming and other changes in HSCs (Miyawaki et al., 2026; Montazersaheb et al., 2022a). Genome-wide DNA methylation analyses reveal that aging is linked to hypermethylation and reduced activity of genes associated with lymphoid cells. Meanwhile, many genes that define HSC identity, including those related to myeloid-biased output, show relative hypomethylation and higher expression levels, reflecting a shift toward myeloid lineage (Yanai et al., 2025). Overall, these age-related declines in HSC function cause increased myeloid bias, reduce lymphocyte numbers, and deterioration of the bone marrow niche, with significant medical consequences. Clinically, hematopoietic aging impairs T- and B-cell responses, thereby increasing vulnerability to infections and reducing vaccine responses.

## Innate immunosenescence

Age-related decline and dysregulation of the innate immune cells impair the initiation and coordination of the host immune response to infection, cancer, or vaccines (Shaw et al., 2013; Lewis et al., 2022). Aged innate immune cells are known to exhibit reduced expression or impaired Toll-like receptor (TLR) signaling, thereby delaying or weakening innate immunity (Panda et al., 2010). Mechanistically, aging directly impacts the response of each innate immune cell. Macrophages and neutrophils show significantly reduced chemotaxis, impaired phagocytosis, and decreased oxidative burst. Macrophages shift toward M2 anti-inflammatory phenotypes in certain tissues, thereby reducing their antigen-presenting ability (Wong et al., 2017; Wang Z. et al., 2025). Consequently, there is reduced dendritic cell migration to lymph nodes and an impaired ability to prime T cells in aged individuals. Lastly, aged NK cells exhibit decreased NK cell-mediated cytotoxicity and cytokine production (Shen et al., 2022; Allen et al., 2020). In addition, aged innate immune cells produce higher basal levels of pro-inflammatory cytokines, including IL-6, TNF-α, and IL-1β, and CRP (Teissier et al., 2022). These age-associated cellular defects and low-grade systemic inflammatory processes contribute to disease progression and poor vaccine responses among elderly individuals. During immunization, innate immunity critically plays a role in vaccine efficacy (Shen et al., 2022). This includes a wide range of mechanisms, such as pro-inflammatory activation, phagocytosis, cytokine production, and antigen presentation, that activate T- and B-cell immunity (Levitz and Golenbock, 2012; Kim et al., 2024). Furthermore, recent research has shown that vaccines can induce innate immune memory via epigenetic and metabolic alterations, highlighting the supportive role of innate immunity in protection post-vaccination. However, aging has been shown to negatively affect trained immunity by inducing a hyperreactive state that leads to tissue damage (Damani-Yokota and Khanna, 2025; Hellgren et al., 2024; Kaufma et al., 2018; Ziogas et al., 2025). In this section, we discuss the impact of age on macrophages, neutrophils, dendritic cells, and NK cells.

## Effect of aging on monocytes, macrophages, and neutrophils

Monocytes, macrophages, and neutrophils are central effector cells of the innate immune response due to their diverse functions (Chiu and Bharat, 2016). Aging results in a plethora of phenotypic and functional changes in these cells. The early effect of aging starts in the bone marrow cells, where a significant reduction of monocytes was found among older individuals (Zhang et al., 2023). Aging in monocytes or monocyte-derived macrophages is associated with reduced phagocytosis, elevated levels of senescent markers, reduced migration and chemotaxis, increased production of inflammatory cytokines, diminished autophagy, and reduced or defective TLR expression. An age-related decrease in TLR gene expression and reduced cytokine production in response to activation of TLR2, TLR3, TLR4, TLR5, TLR6, and TLR9 in splenic and peritoneal macrophages has been reported by several studies (Wang Z. et al., 2025; Shen et al., 2022). These reports indicate that macrophages from aged mice exhibit age-related declines in TLR signal transduction through the MAP and JNK kinase pathways. Such age-related effects on splenic and peritoneal macrophages were suggested to be due to a reduction in LPS-induced phosphorylation of the p38 and JNK mitogen-activated protein kinases (MAPKs) (Duong et al., 2021). Similarly, in humans, the function of TLRs in monocytes decreases with age, resulting in reduced cytokine production among older adults. For example, monocytes from humans aged 65 and older exhibit diminished TLR1/2 function and produce lower levels of IL-6 and TNF-α in response to TLR1/2 stimulation (Panda et al., 2010; Duong et al., 2021; Shaw et al., 2011). In addition, monocyte-derived macrophages from elderly individuals express lower levels of TLR3 (Shaw et al., 2011). Given TLRs’ role in initiating cellular immune activation and function, such defects could lead to a poorer immune response during infection and vaccination in older individuals. Furthermore, analysis of macrophage proportions revealed an increased frequency of CX3CR1+ macrophages and a reduced frequency of Ly6C+ macrophages in old mice compared to young ones (Burgess et al., 2019). The alteration in these two markers suggests a skewing towards a more anti-inflammatory, pro-angiogenic macrophage phenotype in older individuals. Neutrophil counts in healthy young and elderly individuals showed no difference at baseline. However, evidence suggests that with aging, the expansion of circulating CD16^+^CD62L+ immunosuppressive neutrophils occurs and is associated with reduced superoxide production and phagocytosis (Van Avondt et al., 2023). The underlying basis of these aging-related neutrophil phenotypes is complex and linked to multiple factors, including cell-intrinsic alterations, changes in neutrophil local microenvironments, and increased circulating pro-inflammatory mediators with age. Aging impairs neutrophil functions such as migration and phagocytosis but increases Neutrophil Extracellular Traps (NET) secretion, leading to chronic inflammation and reduced immunity in older adults (Wang Z. et al., 2025). Furthermore, neutrophils from elderly individuals exhibited a high rate of early apoptosis. These apoptotic neutrophils lose their receptor-dependent inflammatory responses and are impaired in phagocytosis, degranulation, and Reactive Oxygen Species (ROS) production. Aged neutrophils show no differences in the expression of the IL-8 receptor (CXCR2) and the LPS receptor (TLR4) compared with young individuals; the production of ROS and NET formation triggered by IL-8 and LPS is lower, indicating decreased sensitivity to these stimuli (Torfs et al., 2026; Ozcan and Boyman, 2022). In contrast, other studies indicate that neutrophils in elderly individuals produce more spontaneous ROS, which is linked to chronic inflammation. Additionally, ROS production is crucial for NET formation and NETosis (Hazeldine et al., 2014). These functional deficiencies and excessive neutrophil responses associated with aging may partly explain the cytokine storm or chronic inflammation observed in the elderly. An additional layer of complexity in innate immunosenescence is the heightened production of proinflammatory cytokines (IL-1β, IL-6, TNF, and CRP) by macrophages and neutrophils in elderly individuals (Tylutka et al., 2024). Such a proinflammatory cytokine storm was reported to be the leading cause of the observed immunopathology in COVID-19 (Fara et al., 2020; Mahmoud et al., 2023; Montazersaheb et al., 2022b). Such heightened levels of proinflammatory cytokines could potentially lead to chronic inflammation, therefore increasing the risk of cardiovascular diseases, sarcopenia, and progression of respiratory tract infection and complications (Ferrucci and Fabbri, 2018). However, a deeper understanding of the underlying mechanisms of heightened proinflammatory cytokine production, in relation to defective TLR stimulation, will help restore the innate immune balance between cellular activation and cytokine-mediated responses.

## Aged dendritic cells (mDC and pDC)

Any prophylactic, therapeutic, or vaccinal agent targeting T- and B-cell responses relies heavily on dendritic cell (DC) activation and function (Borchers et al., 2013). Clear evidence has been reported on the importance of DC in T cell activation and function following both vaccination and natural infections (Boyoglu-Barnum et al., 2019; Cham et al., 2020; Chen et al., 2016). While myeloid DC (mDC) and plasmacytoid DC (pDC) play distinct roles, the functions of both DC subsets are affected by aging (Agrawal and Gupta, 2011; Reitsema et al., 2024). Aging leads to a significant reduction in the numbers of mDCs and pDCs and a decrease in CD86 surface expression in older individuals (Jing et al., 2009; Cham et al., 2021). Several other studies have reported an age-related defect in LPS-induced IL-12 production in mDCs, abrogation of mDC migration, and a decline in the ability of antigen-pulsed DCs to stimulate T cell proliferation, resulting in decreased IFN-γ responses (Cham et al., 2021; Jyono et al., 2010). Further studies using a transwell assay showed decreased mDC phagocytosis and migration toward CCL19 or SCF-1, indicating a defect in antigen-presenting function in older adults (Chougnet et al., 2015; Vora et al., 2016). On the other hand, pDCs from older individuals exhibited decreased IFN-α production upon stimulation (Kim et al., 2024), which could be associated with the observed TLR defect in aging. However, stimulation of monocyte-derived DCs from older individuals resulted in increased production of TNF-α and IL-6 compared with those from younger individuals (Agrawal et al., 2012). Although the extent of DC immunosenescence remains unclear, understanding the mechanisms underlying age-associated changes in both mDCs and pDCs will be necessary to improve immune response among the elderly. This could potentially resolve the observed age-associated abrogation of infection outcomes, dysfunctional vaccine responses, and a decline in protection among elderly individuals.

## Aging and NK cell number and function

As discussed above, hematopoietic aging leads to myeloid-biased differentiation and reduced lymphopoiesis, resulting in fewer lymphocytes. However, aging in humans has been shown to be associated with an increase in the absolute number and percentage of CD3^−^CD56^+^CD16^+^ NK cells in peripheral blood (Chidrawar et al., 2006). Several studies reported that in the elderly, there is an increase in the number and a redistribution of NK cell subpopulations. The frequency and composition of NK cells change significantly, with a general shift toward a more immature and less functional phenotype (Chidrawar et al., 2006; Chen S. et al., 2024). These reports show a decrease in CD56bright NK cells, an increase in immature NK cells, and an increase in CD56dim NK cells (Gounder et al., 2018). The CD56bright NK cell subset primarily secretes cytokines such as IFN-γ and TNF-α, whilst the CD56dim NK cells are known for their cytotoxic function. However, despite the increase in CD56dim NK cells, aged NK cells exhibit a significant decline in cytotoxic activity (Shehata et al., 2015). The terminally differentiated CD56dim NK cell population shows increased expression of HLA-DR and CD95 (Fas) on its surface (Kust et al., 2024), along with reduced levels of CD69 (a C-type lectin and early activation antigen) compared to NK cells from young individuals (Camous et al., 2012). There is consensus that NK-cell immunosenescence impairs their functional capabilities, and the underlying mechanism of this intrinsic reduction in cytotoxic activity remains poorly understood (Gounder et al., 2018; Hazeldine and Lord, 2013). Immunologically, NK cell-mediated cytotoxicity and cytokine secretion are the primary mechanisms by which NK cells contribute to host immune response. Therefore, age-related impairment of NK cells could contribute to delayed control of infection and poor vaccine response.

## Adaptive immunosenescence

Considering the role of T and B cells in coordinating the host immune response to infections, cancer, and vaccines, understanding how aging impacts these adaptive immune cells is crucial. Adaptive immunosenescence is the age-related decline in the number and function of T and B lymphocytes (Goyani et al., 2024). This process results from both intrinsic cellular changes and external factors. The primary factors include decreased lymphopoiesis, thymic involution, reduced cellular diversity, and a shift from naive to memory T and B cells. Other key factors contributing to T and B cell aging include mitochondrial dysfunction, genetic and epigenetic changes, loss of proteostasis, a reduced TCR and BCR repertoire, an imbalance between naïve and memory cells, and decreased effector plasticity (Goyani et al., 2024; Levitz and Golenbock, 2012; Kim et al., 2024; Santamaria and Irla, 2026). These factors may lead to a reduction in naive T cells, accumulation of senescent T and B cells, T cell exhaustion, reduced B-cell diversity, and lower antibody production. The clinical consequences of adaptive immunosenescence may include increased susceptibility to infections and cancers, inflammaging, poor vaccine response, and reduced protection.

## Age-related decreased T-Lymphopoiesis and thymic involution

In humans, aging leads to a significant decrease in T lymphopoiesis (the production of new T cells), primarily due to HSC dysfunction and thymic involution (Nikolich-Zug and ich, 2008). The age-related decline in the production of new naïve T cells likely begins in the bone marrow, where HSCs that give rise to lymphocyte progenitors exhibit age-related functional abnormalities (Lai and Kondo, 2008). Earlier reports have shown that aged HSCs accumulate mutations and undergo epigenetic changes that suppress lymphoid-specific genes such as EBF1 and PAX5, while activating myeloid pathways. Other reports have suggested that the loss of HSCs’ ability to generate T cells is due to an irreversible intrinsic loss of lymphoid potential (Goyani et al., 2024; Weiskopf et al., 2016; Wells et al., 2024). Age-associated impairment of multipotent HSC diversity and differentiation potential results in reduced polymorphism and fewer CD62L+ HSCs. Such a reduction in CD62L+ HSCs is associated with decreased lymphoid differentiation and a general decline in immune function, rather than directly enhancing lymphocyte homing to secondary lymphoid organs (Kohn et al., 2012; Kapitza et al., 2023). The reduced ability of aged HSCs to generate lymphocytes is also suggested to result from increased expression of hypoxia-inducible factor 1-alpha (Tang et al., 2022). In addition to age-related HSC impairment, thymic involution is another key factor contributing to reduced T cell numbers and function in the elderly. Thymic involution is the progressive, age-related shrinking of the thymus gland characterized by a loss of epithelial cells, reduction of cortical-medullary junctions, and increased fatty tissue accumulation. Thymic involution results from various factors, including hormonal changes and chronic inflammation, leading to functional alterations in the hematopoietic and stromal compartments (Santamaria and Irla, 2026; G et al., 2012). These gradual changes disrupt the interaction between developing T cells and thymic epithelial cells, which is essential for maintaining thymic function (Reis et al., 2015). In aged humans, medullary thymic epithelial cells in the thymus exhibited reduced expression of forkhead box N1 (FOXN1), a transcription factor essential for thymic T cell development (G et al., 2012; Reis et al., 2015). Mechanistically, reduced expression of FoxN1 results in the loss of thymic architecture and, subsequently, low secretion of IL-7, a vital cytokine for thymocyte survival and proliferation. Other studies suggest that aging leads to the replacement of functional thymic tissue with adipocytes and fibroblasts, a process that does not support T cell development (Fernandez Maestre et al., 2025; Velardi et al., 2021; Choi et al., 2025). These age‐related thymic involution factors, along with others such as transcription factors, microRNAs, growth factors, and cytokines, lead to a drastic decline in the number of naïve T cells exported to the peripheral blood (Cham et al., 2021; Wang et al., 2022).

## Effect of aging on T cell number and function

T cell aging is one of the most crucial aspects of immunosenescence, given its impact on overall immune responses (Shen et al., 2024). Aging in CD4^+^ and CD8^+^ T cells involves declines in naïve T cell production, TCR repertoire, homing markers, co-stimulatory receptors, and cytokine production, and increases in inhibitory and exhaustion markers (Moro-Garcia et al., 2013; Jo et al., 2023). Profiling studies have demonstrated age-related changes in the composition and function of CD4^+^ and CD8^+^ T cells. With age, both the total number and percentage of CD4^+^ and CD8^+^ T cells within the CD3^+^ T cell population decreased significantly in the peripheral blood (Shen et al., 2024; Chang et al., 2024; Li et al., 2019). However, the percentages of Central Memory T cells, Effector Memory T cells, and Effector T cells within the CD4^+^ and CD8^+^ T cell subsets increased with age (Shen et al., 2024). When examining different memory T cell subsets, only percentage changes can be detected, with no apparent changes in absolute cell numbers, partly due to differences in the subsets analysed. Characterization of changes in the co-stimulatory molecules CD28 and FAS (CD95) across T cell subsets with age shows that the percentages of CD28^−^and CD95^+^ T cell subsets increased with age in both CD4 and CD8 populations. But only the CD4^+^ CD28^−^ T cells showed an increase in absolute numbers (Moro-Garcia et al., 2013; Weng et al., 2009). Within the CD4 T cell subset, studies on the Th1/Th2 balance are sometimes contradictory, with some reports describing a Th2-skewed response and others a Th1-skewed response in older adults. However, both Th1 and Th2 cells are largely maintained in the circulation in the elderly (Uciechowski et al., 2008; Cataneo-Pina et al., 2025; Gasparoni et al., 2003). The proportion of Th17 cells increases in the peripheral blood of older adults. Although the frequency of memory Th17 cells might decrease, the differentiation of naïve CD4^+^ T cells into Th17 cells increases with age. Given that Th17 cells secrete pro-inflammatory cytokines, their age-related increase likely contributes to inflammaging (Lee et al., 2011). Tregs are generally reported to increase in frequency with age, thereby contributing to immunosuppression. While the proportion of naïve Tregs decreases, memory Treg cells increase, thereby increasing the Treg/Teff ratio in the memory CD4^+^ T cell compartment of older adults, which correlates with increased immune tolerance (Churov et al., 2020). Consequently, experimental approaches that selectively deplete Tregs have been shown to improve vaccine immunogenicity (Batista-Duharte et al., 2022). Functionally, naïve CD4 T cells are shown to be impaired in both *in vitro* and *in vivo* settings (Luckheeram et al., 2012). Moreover, a key consequence of TCR activation in naïve CD4 T cells is the secretion of IL-2, an essential cytokine for T cell survival and growth. Naïve CD4 T cells from older individuals produce less IL-2 and show decreased proliferation upon stimulation with antigen-presenting cells (Salam et al., 2013; Swain et al., 2005). This may explain their impaired ability to generate Th1 or Th2 responses, which can be partially rescued by external IL-2, suggesting these cells still respond to IL-2 (Swain et al., 2005; Ho et al., 2024). Effector cells derived from aged naïve CD4 T cells also display lower levels of differentiation and activation markers, such as CD25 and CD62L. The defects in aged CD4^+^ T cells, including reduced proliferation and cytokine secretion, were also seen in memory cells derived from aged naïve CD4 T cells (Haynes and Swain, 2006; Harpaz et al., 2017). This suggests a diminished capacity to generate functional memory, which could not be corrected even with external IL-2 treatment (Swain et al., 2005). Additionally, although a significantly increased number of CD69^+^CD4^+^ T cells were observed in aged individuals, a significantly decreased production of IFN-γ by aged CD4^+^ T cells was reported (Jo et al., 2023; Ho et al., 2024).

Age-CD8^+^ T cells undergo a series of morphological and molecular changes, with alterations in cell-surface markers serving as key indicators for identifying senescent CD8^+^ T cells (Dock and Effros, 2011). Age-associated CD8^+^ T cells have been shown to have reduced expression of the co-stimulatory molecules CD27 and CD28. Studies have demonstrated that the decreased expression of both CD27 and CD28 suppresses telomerase activity by downregulating the human telomerase RNA component, thereby compromising telomerase integrity and accelerating the aging process (Weng et al., 2009; Patrick et al., 2019; Griffiths et al., 2013). Additionally, the increased expression of KLRG-1 and CD57 further distinguishes aged CD8^+^ T cells. Furthermore, increased CD57 expression, higher p53β expression, and lower Δ133p53 on aged CD8^+^ T cells decrease proliferation (Mondal et al., 2013). Moreover, aging in CD8^+^ T cells is characterized by reduced functional capacity and exacerbated T cell exhaustion. The elevated expression of PD-1, TIM-3, and LAG-3 on aged CD8^+^ T cells may explain the increased rate of T-cell apoptosis in older adults (Nikolich-Zug and ich, 2008; Banica et al., 2021). Furthermore, as mentioned above, the activity of antigen-presenting cells, such as dendritic cells and macrophages, is significantly reduced, while the activity of immunosuppressive cells, including regulatory T cells and myeloid-derived suppressor cells, is elevated (Panda et al., 2010; Duong et al., 2021; Shaw et al., 2011). In the periphery, circulating CD8^+^ T cells in the elderly exhibit diminished TCR diversity, leading to a loss of specificity against pathogens and cancers. Furthermore, aging suppresses TCR signalling and metabolic reprogramming, therefore affecting CD8^+^ T cell activation (Fernandez Maestre et al., 2025; Velardi et al., 2021; Choi et al., 2025). Some studies suggest that in elderly mice, CD8 T cells may exhibit faster cytotoxic responses, but this often comes at the expense of decreased viability (Griffiths et al., 2013). Other research indicates that senescent CD8^+^ T cells can lose their ability to perform multiple functions and have inherent defects in lysing target cells, despite producing high levels of cytotoxic proteins. Age-CD8 T cells release pro-inflammatory cytokines, such as TNF-α and IFN-γ, along with a plethora of other cytokines and chemoattractants. In addition, age-CD8 T cells secrete GZMK, which can cleave extracellular substrates and promote inflammation. These events set the stage and contribute to the development of inflammaging (Griffiths et al., 2013; Banica et al., 2021; Nikolich-Zugich et al., 2012; Xin et al., 2025).

## Effect of aging on B-cell and humoral response

As discussed in the section above, aging leads to myeloid-biased differentiation and decreased lymphopoiesis, thereby reducing the generation of new naive B cells (Rodriguez-Zhurbenko et al., 2019). The effect of aging was observed in the early development of the B cell lineage (Riley, 2013; Lee J. L. et al., 2022). It is well known that pro- and pre-B cell precursors express two specific cell-surface markers, such as CD10 and the IL-7Rα receptor (Clark et al., 2014). In the presence of IL-17, B cells undergo rearrangement of the immunoglobulin (Ig) heavy chain genes, a process that involves the RAG-1 and RAG-2 genes. Once rearrangement is successful, these cells enter the pre-B cell stage, characterized by expression of the pre-B cell receptor, along with the transmembrane signaling molecules Igα and Igβ, followed by rearrangement of the Ig light-chain genes (Novobrantseva et al., 1999; Milovanovic et al., 2010). After completing Ig gene rearrangement, RAG gene expression decreases, and CD10^+^ immature B cells in the bone marrow lose IL-7Rα expression, begin to express CD5, and start producing IgM. This demonstrates the importance of IL-7 and IL-7Rα in the early development of the B cell lineage (Agrawal and Gupta, 2011; Alter-Wolf et al., 2009; Revilla et al., 2012; Kaiser et al., 2023). However, aging in humans is reported to significantly alter the IL-7 signalling within the bone marrow. The primary source of IL-7 in the bone marrow is stromal cells (Nguyen et al., 2017). As individuals age, these cells often become senescent or dysfunctional, resulting in reduced IL-7 secretion. Additionally, elevated levels of TNF-α and TGF-β, driven by chronic inflammation or inflammaging in the bone marrow, impair stromal cells’ ability to secrete IL-7. Furthermore, elderly individuals exhibit lower IL-7Ra expression on B cells, thereby diminishing their capacity to survive and proliferate (Nguyen et al., 2017; de Mol et al., 2021; Ma et al., 2019). These B progenitors exhibit reduced activation of key survival and proliferation pathways, particularly the JAK/STAT5 and PI3K/Akt pathways, which are essential for survival through the IL-7 receptor (Lee J. L. et al., 2022; Kaiser et al., 2023; Frasca and Blomberg, 2009). Other studies indicate that although IL-7Rα expression levels on early B-cell progenitors may not always decline, the signalling effectiveness decreases, resulting in impaired B-cell development. This significant age-related decline in mature B cells is driven not only by reduced production of new B cells but also by alterations in the microenvironment that supports their survival in the periphery (Kaiser et al., 2023; Nguyen et al., 2017). The TNF ligands, BAFF and APRIL, are critical for the survival of peripheral mature B cells, and their levels have been reported to decrease in the plasma of older adults. In addition to the age-associated reduction in B cells, aging significantly alters B cell subsets (Corfe and Paige, 2012). There is a well-documented decrease in the proportion of naïve B cells and a relative increase in memory B cells, leading to a restricted repertoire (Riley, 2013). A decreased class-switched memory IgD-CD27^+^ B cells, and the accumulation of (CD11c+Tbet+CD21^−^) age-associated B cells are reported in elderly individuals. These age-associated B cells (ABCs) are often activated through TLR7 and TLR9 pathways, resulting in antibody secretion upon TLR stimulation rather than BCR stimulation (Ma et al., 2019; Lamprinou et al., 2026; Pinto et al., 2023). Meanwhile, the increased sensitivity of ABCs to TLR7/9 stimulation is thought to cause greater autoantibody production (Rubtsov et al., 2013), which may lead to autoimmune diseases in the elderly. Although B cells in older individuals exhibit diminished responses to infections, they also show higher baseline TLR activity and produce more pro-inflammatory cytokines, such as TNF-α and IL-6, thereby contributing to inflammaging (Nguyen et al., 2017).

The B cell activation pathway involves naïve B cells encountering specific antigens via BCR, leading to proliferation, differentiation into antibody-secreting plasma cells, and the formation of memory cells. Activated B cells differentiate into effector cells that secrete IgM and then undergo somatic hypermutation and class switching from producing IgM to IgG (Rodriguez-Zhurbenko et al., 2019; Choi et al., 2012). This process typically requires two signals: antigen binding and CD4^+^ T cell help (particularly T follicular helper cells), both of which are affected by aging (Frasca and Blomberg, 2009; Han et al., 2003). Aging diminishes T follicular helper (Tfh) cell function, leading to weaker germinal center responses, reduced high-affinity antibody production, and compromised vaccine immunity (de Lisio et al., 2025). These Tfh cells are a specialized subset of CD4^+^ T cells that provide essential help to B cells within lymphoid follicles and germinal centers, facilitating high-affinity antibody production, switching, and the generation of long-lived memory B cells and plasma cells. Tfh cells interact closely with B cells via CD40L-CD40 and IL-21 to drive selection and differentiation. Within germinal centers, Tfh cells promote B cell proliferation, somatic hypermutation, and class switch recombination, and selectively provide survival signals to B cells presenting the highest-affinity antigens, thereby driving affinity maturation (de Lisio et al., 2025; Gustafs et al., 2018; Deenick and Ma, 2011). Moreover, B cells from elderly individuals have decreased responsiveness to stimuli such as TLRs and CD40, leading to reduced proliferation and lower-quality antibody production (Khan et al., 2026). Aged B cells show decreased levels of molecules like activation-induced cytidine deaminase (AID) and the transcription factor (E47) that are essential to switch from IgM to IgG/IgA. Aging substantially impairs the transcription factor E47 in activated B cells, primarily due to reduced mRNA stability (Frasca et al., 2008). This decline results in lower AID levels, thereby impairing their capacity to generate high-affinity IgG antibodies (Han et al., 2003; Goenka et al., 2014). Moreover, aging significantly reduces Fc-mediated effector functions, particularly antibody-dependent cellular cytotoxicity (ADCC) and antibody-dependent cellular phagocytosis (ADCP) (Sievers et al., 2025). This decline is mainly due to immunosenescence, which involves decreased NK cell activity and changes in IgG subclass distribution and glycosylation patterns. Although total IgG levels often stay the same or rise in older adults, the quality of the antibody response deteriorates. This is evidenced by decreased IgG1 functionality and increased levels of pro-inflammatory agalactosylated IgG, both of which hinder the immune system’s capacity to eliminate infected cells or circulating pathogens (Sievers et al., 2025; Chen X. et al., 2025; Lakerveld et al., 2023). Overall, there is consensus that aging leads to a reduction in circulating plasma B cells, a decrease in class-switched B cells, and impaired antibody affinity maturation, which can undermine vaccine immunogenicity and reduce protection following vaccination.

## Inflammaging

The age-related phenomenon of inflammaging is characterized by low-grade inflammation and is not necessarily caused by infection, or clinically active diseases (Franceschi and Campisi, 2014). Healthy older adults exhibit elevated levels of inflammatory markers, such as TNF-α, CRP, IL-6, IL-1, IL-18, and IL-1β, compared with healthy younger adults (Singh and Newman, 2011; Mathot et al., 2025). The age-associated increase in inflammatory markers has been attributed to an imbalance of inflammatory cytokines and cellular senescence (Franceschi and Campisi, 2014). Macrophages are central drivers of inflammaging, and their dysregulation with age is a primary contributor. Age-related alterations in macrophage phenotypes involve a skewed balance toward M1 macrophage polarization. These M1 macrophages secrete a variety of inflammatory and bactericidal mediators, including IL-6, TNFα, IL-1β, IL-12, and ROS, all of which are significantly elevated among elderly individuals. This is also accompanied by upregulation of the MCP-1 gene and key glycolytic genes such as hexokinase-2 and phosphofructokinase (Wang Z. et al., 2025; Shen et al., 2022). Similarly, neutrophils are key contributor of inflammaging through increased NETosis and persistent activation (Torfs et al., 2026; Ozcan and Boyman, 2022). While NK cells, T cells, and other immune cells also contribute to inflammaging (Fara et al., 2020; Mahmoud et al., 2023; Montazersaheb et al., 2022b). In B cells, aging favours the accumulation of CD11c+Tbet+CD21^−^age-associated B cells. These age-associated B cells act as autoantibody-producing cells and pro-inflammatory cells that secrete significantly elevated levels of TNF-α, IL-6, IL-12, and IFN-γ, therefore significantly contributing to inflammaging (Ma et al., 2019; Lamprinou et al., 2026; Pinto et al., 2023). Inflammaging results from both intrinsic aging processes and external environmental factors that together influence cellular and molecular pathways. Several studies have reported different triggering factors of inflammaging (Karpuzoglu et al., 2025). Studies exploring internal age-related factors contributing to inflammaging indicate that immune cells accumulate with age due to repeated cellular stress, telomere shortening, DNA damage, and oncogenic signaling that can trigger inflammaging. These age-cells develop a distinct phenotype characterized by permanent cell-cycle arrest and the Senescence-Associated Secretory Phenotype (SASP), which includes pro-inflammatory cytokines (IL-6, IL-1β, TNF-α), chemokines, growth factors, and proteases, thereby driving local and systemic inflammation (Cham et al., 2021; Karpuzoglu et al., 2025; Baechle et al., 2023). Another factor is the role of mitochondria, which serve as key centres of cellular metabolism and innate immune signaling (Zhang et al., 2025). As we age, mitochondrial dysfunction becomes widespread, characterized by reduced oxidative phosphorylation, increased ROS production, and the release of mitochondrial damage-associated molecular patterns (mtDAMPs), including mitochondrial DNA (mtDNA) and cardiolipin. When mitochondria are damaged, they release mtDNA and ROS, which act as DAMPs and activate innate immune sensors like the NLRP3 inflammasome, leading to cytokine production (Zhang et al., 2025; Grazioli and Pugin, 2018; Hruby and Higuchi-Sanabria, 2025). Additionally, aging is associated with dysregulation of complement activation, leading to increased production of small protein fragments called anaphylatoxins, mainly C3a, C4a, and C5a. These fragments bind to receptors on immune cells, triggering production of proinflammatory cytokines and chemokines. Recent evidence shows that gut microbiota can contribute to inflammaging (Khazi and Ahmad, 2025; Zheng et al., 2022; Armento et al., 2021). Aging can alter the gut microbiota, resulting in increased lipopolysaccharide secretion, which activates nuclear factor-kappa B (NF-κB). These findings suggest that LPS from gut bacteria contributes to age-related inflammation. It is well known that NF-κB activation increases IL-6 expression, which may explain the observed increase in inflammation among the elderly (Khazi and Ahmad, 2025; Heston et al., 2023). Furthermore, chronic viral infections such as cytomegalovirus (CMV) and Epstein-Barr virus (EBV) contribute to inflammaging through persistent immune activation and disruption of immune cell regulation (de Melo Silva et al., 2020). The host’s constant effort to control these chronic viral infections results in elevated cytokine (IL-6, IL-1β, TNF-α) production, thereby contributing to inflammaging (Muller and Di Benedetto, 2024). Additionally, persistent CMV infection primarily drives T-cell differentiation toward senescence and induces B-cell dysfunction. CMV infection induces a massive, persistent expansion of CMV-specific CD8^+^ T cells, leading to massive cytotoxic granule release and cytokine production, including perforin, granzymes, TNF-α, and IFN-γ. Such a chronic antigenic stimulation leads to high expression of PD-1 and TIGIT and diminished cytotoxic capacity (Muller and Di Benedetto, 2024; Pachnio et al., 2016). This may explain reports that CMV-positive individuals often exhibit lower vaccine-specific antibody response effectiveness. Furthermore, CMV infection increases intracellular TNF-α expression in B cells, which correlates with decreased B-cell function and reduced percentages of switched memory B cells. In older adults, CMV seropositivity is associated with higher amounts of serum TNF-α and has been shown to have a negative effect on vaccine-specific antibody responses. (Chen et al., 2018; Hu et al., 2025; Merani et al., 2017). Overall, inflammaging profoundly alters T- and B-cell phenotype and function. Elevated age-associated levels of TNF-α, IL-6, and IL-1β drive T cell dysfunction by creating a hostile environment that induces T cell senescence and exhaustion. This process reduces the number of naive T cells, increases memory T cell dysfunction, and fuels a vicious cycle of persistent inflammation, thereby reducing vaccine efficacy. Moreover, the chronic inflammatory environment during inflammaging reduces expression of activation-induced cytidine deaminase in B cells, a key enzyme for class-switch recombination and somatic hypermutation, thereby leading to lower-affinity antibody production and abrogating vaccine response (Frasca et al., 2014).

## Impact of immunosenescence and inflammaging on vaccine response

**FIGURE 2:**
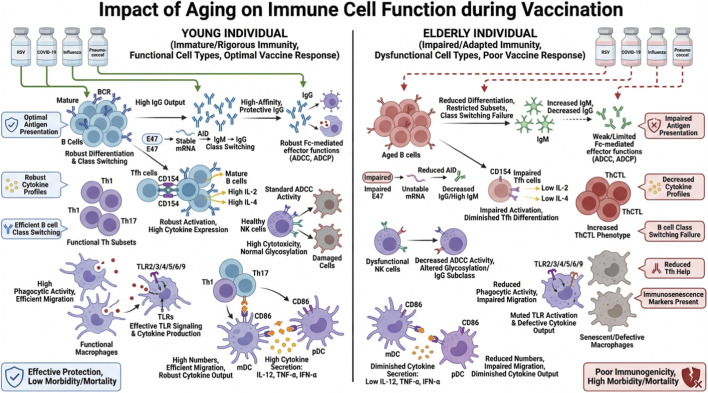
Impact of aging on immune cell function during vaccination: An illustration of the impact of aging on reducing vaccine response due to reduced B cell numbers and poorer antibody quality, with declines in transcription factors such as E47 and AID that affect high-affinity IgG production, weakening Fc functions like ADCC and ADCP, and impairment of CD4^+^ T cells and APCs function.

As discussed in the previous sections, aging reduces naive B-cell numbers and contracts the BCR repertoire, leading to a less diverse pool of B-cell specificities. Aging leads to the formation of smaller and fewer germinal centers, which are essential for producing high-affinity antibodies and memory B cells (Han et al., 2003; Goenka et al., 2014). This reduction is driven by defects in follicular dendritic cells (FDCs) and a reduced T follicular helper (Tfh) cell response. Age-related intrinsic T cell defects in aged naïve CD4^+^ T cells, such as reduced ability to activate and migrate into lymph nodes, can also lead to suboptimal Tfh cell differentiation, which is essential in vaccine response (de Lisio et al., 2025; Gustafs et al., 2018; Deenick and Ma, 2011). Age-associated reductions in TCR and BCR repertoire diversity are a hallmark and a core component of immunosenescence, directly contributing to diminished vaccine responsiveness in older adults (Allen et al., 2020). A decreased TCR repertoire, or reduced diversity among T-cell clones, directly correlates with compromised antigen-specific functionality, leading to reduced vaccine responsiveness. A reduced T-cell repertoire means fewer unique T-cells are available to recognize the different components of a vaccine. Age-associated declines in TCR diversity led to reduced responsiveness, particularly evident in the elderly’s impaired ability to mount robust responses to influenza vaccines (Oh et al., 2019). Studies on COVID-19 indicate that a decreased CD8^+^ T-cell repertoire correlates with higher disease severity after infection, even among vaccinated individuals (Brunetti et al., 2025; Almendro-Vazquez et al., 2023). On the other hand, aging is associated with increased clonal expansion of antigen-experienced cells, thereby decreasing overall BCR repertoire diversity. The diminished B cell diversity often results in a reduced germinal center response, a critical process for creating high-affinity antibodies and long-term memory B cells. Reduced BCR diversity is often accompanied by lower levels of somatic hypermutation. Moreover, A decreased repertoire impairs the generation of long-lived memory B cells. Studies show a correlation between lower BCR repertoire diversity (particularly in IgM) and poor vaccine-specific antibody responses to vaccines like the pneumococcal vaccine (Hoehn et al., 2016; Ghraichy et al., 2018). (Han et al., 2003; Goenka et al., 2014). During vaccination, aging affects not only antibody production and affinity but also the duration of protective immunity (Frasca et al., 2011). Such effects likely result from combined defects in T cells, B cells, and other antigen-presenting cells. As mentioned earlier, B cells’ capacity to mount an optimal antibody response to vaccines diminishes with age. Aging has been shown to markedly reduce Ig class switch recombination, AID levels, E47 transcription factor levels, and somatic hypermutation. An age-related progressive decline in B cell numbers, together with decreases in CSR, AID, and E47 functional factors, can result in fewer antibody-secreting plasma cells and reduced affinity antibody production (Han et al., 2003; Goenka et al., 2014). In the elderly, Ig production shifts toward decreased IgG and increased IgM, indicating reduced antibody class-switching. Moreover, the antibodies generated in older individuals are less protective than those generated in younger individuals, due to age-associated alterations in the B cell repertoire (Frasca et al., 2008). There are conflicting reports that total antibody levels might remain stable in the elderly compared to young individuals, but antibody quality declines, with a decreased ability to produce high-affinity antibodies. Aging reduces the functionality and diversity of memory B cells, impairing their ability to respond to reinfection after vaccination, thereby explaining the short-term protection observed in elderly individuals (Mink et al., 2024). Additionally, inflammaging has been shown to elevate TNF-α- producing aged B cells, which significantly reduce AID, thereby hindering class-switch recombination and somatic hypermutation while promoting autoantibody production (Ma et al., 2019; Lamprinou et al., 2026; Pinto et al., 2023). On the other hand, poor durability and low-affinity antibodies among older adults are associated with reduced T follicular helper (Tfh) cell function and decreased germinal center B-cell responses. As discussed above, aging leads to diminished Tfh cell numbers and reduced secretion of IL-21, IL-4, and CD40L, which are crucial to initiating an effective germinal center B cell response. This directly reduces antibody affinity and the generation of long-lived plasma cells, leading to lower-quality, less durable antibody responses in older adults (Jo et al., 2023; Ho et al., 2024). In addition to the age-related defect and diminished Tfh cells, aged germinal center B cells exhibit decreased somatic hypermutation and class-switch recombination, resulting in lower-affinity antibodies and reduced production of long-lived plasma cells and memory B cells. These age-related dysfunctions in Tfh and germinal center B cells have been shown to lead to weaker antibody neutralization capacity and faster waning of immunity in vaccinated older individuals compared to younger adults (Jo et al., 2023; Luckheeram et al., 2012; Ho et al., 2024). Furthermore, studies have shown that aging impairs CD4^+^ T cells’ ability to mount new Th1 responses, which explains why older adults have reduced capacity to produce Th1 cytokines (IL-2, IFN-γ) when challenged with a vaccine (Salam et al., 2013; Swain et al., 2005). Innate immune cells, particularly macrophages and dendritic cells, act as sentinels and key orchestrators in vaccine responses. They phagocytose and present antigens to T cells, initiating adaptive immunity. Dendritic cells display a pro-inflammatory phenotype during aging, with increased IL-6 and TNF-α levels but reduced levels of anti-inflammatory cytokines and type I interferon secretion. This reduces their antigen-presentation capacity, thereby affecting T cell priming and tolerance maintenance (Cham et al., 2021; Jyono et al., 2010). The age-associated effects on macrophages and dendritic cells are linked to epigenetic reprogramming and defective TLR signaling (Alnakhli et al., 2026), further exacerbating age-associated immune dysfunction to vaccine response. Together, these age-related defects in innate and adaptive immune cells lead to poor vaccine immunogenicity and efficacy, resulting in higher morbidity and mortality from viral and bacterial infections post-immunization in the elderly population. In this section, we summarize recent knowledge on the impact of aging on reduced vaccine response against respiratory tract infections. Figure 2 highlights the overall effect of aging on immune dysfunction following an immunization.

## Aging and RSV vaccination

Respiratory syncytial virus (RSV) is an RNA virus in the family Pneumoviridae and is among the most common causes of acute lower respiratory infections in children worldwide (Hon et al., 2023). Over the past decade, RSV infection has emerged as a major global health issue, placing a substantial burden on infants and the elderly (Duan et al., 2025). Recent epidemiological studies estimate over 33 million cases of acute RSV-related lower respiratory tract infections, 3.6 million hospitalizations, and more than 100,000 deaths globally (Li et al., 2022; Gil-Prieto et al., 2025). RSV infection is the leading cause of lower respiratory tract infection in children, commonly presenting as bronchiolitis, characterized by cough and varying degrees of hypoxia due to airway congestion (Kim and Choi, 2024; Munro et al., 2023; Divarathna et al., 2023; Piedimonte and Perez, 2014). Neonates, infants, and the elderly are at higher risk of severe RSV infection (Divarathna et al., 2023). Younger adults, on the other hand, exhibit strong cellular and humoral immunity and are often asymptomatic after a primary RSV infection. However, adults older than 60 years can manifest severe diseases such as pneumonia or bronchitis due to immunosenescence and inflammaging (Abu-Raya et al., 2023). While protective RSV immune effector mechanisms have not yet been clearly defined, some evidence suggests that serum neutralizing antibodies against the virus may correlate with immunity (Boyoglu-Barnum et al., 2019; Liu et al., 2022). Additionally, in elderly individuals, pDCs show reduced IFN-α production following stimulation (Stout-Delgado et al., 2008). In older adults, impaired migration of mDCs and reduced ability to support T cell proliferation and priming were observed, leading to a diminished IFN-γ response. Additionally, innate immunosenescence involves increased production of proinflammatory cytokines such as IL-6, IL-1β, and TNF-α, along with CRP, by innate immune cells (Georgakopoulou and Pitiriga, 2025; Brown et al., 2015; Bergeron and Tripp, 2021). In elderly individuals, RSV-specific serum neutralization titres are inversely associated with risk of reinfection, disease severity, and hospitalization (Walsh et al., 2018). RSV-specific T-cell responses are reported to play a crucial role in controlling disease severity. However, reductions in these responses due to immunosenescence likely contribute to older adults’ susceptibility to severe RSV-associated lower respiratory tract disease (Schwarz et al., 2024; Ackerson et al., 2019). Furthermore, immunosenescence and inflammaging make it more challenging to develop an RSV vaccine for the elderly population. So far, only three RSV vaccines (Arexvy, mResvia, and Abrysvo) are currently available for adults older than 60 years. By targeting the pre-F glycoprotein, these vaccines stimulate the production of neutralizing antibodies, activate cellular immune responses, and promote the formation of memory B and T cells (Awosika and Patel, 2024; Agac et al., 2023). Only a few studies have assessed the host immune response to the novel RSV vaccines. However, reports have highlighted that these newly approved RSV vaccines differ significantly in terms of their ability to induce cellular immunity and neutralizing antibody profiles (Schwarz et al., 2024; Agac et al., 2023). Aged T cells have been shown to respond poorly to RSV infection. This is indicated by decreased T cell proliferation, fewer RSV-specific CD4^+^ and CD8^+^ T cells, and diminished effector function as measured by IFN-γ production (Roumanes et al., 2018; Cherukuri et al., 2013; Salaun et al., 2023). Furthermore, aging worsens B cells’ ability to generate RSV-specific IgG. In older adults, RSV infection results in fewer class-switched B cells and reduced antibody production among vaccinated individuals (Frasca et al., 2020; Fulton and Varga, 2009). All these reports have highlighted the negative impact of age on achieving the targeted post-immunization protection rate. Despite the age-related defect, RSV vaccination in older adults is highly effective, with studies indicating 77%–90% protection against severe RSV-associated lower respiratory tract disease (pneumonia, bronchitis) and 67%–68% protection against acute respiratory disease. (Awosika and Patel, 2024; Agac et al., 2023).

## Aging and influenza vaccination

Influenza virus infection is a highly contagious, airborne disease that leads to acute febrile respiratory illness. The virus belongs to the Orthomyxoviridae family and has three types: A, B, and C. Of these, influenza A is the most prevalent and severe, mainly impacting humans (Rousogianni et al., 2025). Recent outbreaks of H1N1 (swine flu), H5N1 (bird flu), and H7N9 highlight the serious threat influenza viruses pose to human health (Possas et al., 2025). Influenza virus infection can often lead to acute respiratory tract complications, particularly during the winter season (Moghadami, 2017). Routine seasonal influenza vaccination is observed globally, particularly in developed countries. However, several factors have been reported to limit the success of influenza vaccination (Trombetta et al., 2022; Zornoza-Moreno et al., 2025). These factors include age, genetic polymorphisms, host-related factors such as preexisting immunity, and chronic underlying conditions, which may compromise influenza vaccine responsiveness (Zornoza-Moreno et al., 2025). Compared to younger adults, Influenza vaccines provide less protection, therefore leading to influenza-associated hospitalizations among elderly individuals (Smetana et al., 2018). In addition, the vaccine response is worsened, especially when the circulating influenza strains are antigenically variant (Mokalla et al., 2025). In older adults, seasonal influenza antigens and antigenic imprinting create a hierarchical antigenic seniority, where the highest antibody titers are consistently maintained against strains from their youth. There is a marked increase in the prevalence of cross-reactive serum antibodies recognizing both H1N1 and H3N2 components among the elderly (Maltseva et al., 2024). For instance, individuals born in the 1950s–1960s were often first exposed to H1N1 or H2N2, while those born after 1968 were exposed to H3N2, shaping their lifetime response to H3N2 vaccines. Older adults’ responses are influenced by the specific H1N1 or H3N2 subtype that dominated their childhood. Individuals born in this era were primarily exposed to H1N1 or H2N2 pandemic strains early in life, creating a “prime” that provides strong immunity against these subtypes. However, this imprinting makes them more susceptible to severe disease during H3N2 pandemics or seasons, as their memory B cells are biased toward the older strains, leading to weaker neutralizing antibody responses to H3N2 vaccines. (Sicca et al., 2022). Therefore, understanding an individual’s lifetime influenza exposure history is necessary for developing more tailored and effective vaccination strategies. Recent evidence has shown that serum antibody responses following influenza vaccination persist shorter among the elderly population (Young et al., 2017; McElhaney, 2011), suggesting an alternative vaccination strategy to avoid hospitalization.

Given the importance of T cells in influenza vaccine efficacy (Schmidt and Lapuente, 2021), recent studies have shown that defective dendritic cells in the elderly population contribute to the abrogation of T cell responses during immunization (Grolleau-Julius et al., 2008; Fulop et al., 2022). Older adults exhibit a contracted naive repertoire and decreased intralineage diversification, suggesting that older B cells have a reduced ability to generate novel antibody responses. Furthermore, the transcription factor PAX5 is a crucial regulator of B cells and is decreased in mature B cells from elderly individuals. This reduction in PAX5 expression on B cells in the elderly is associated with an increase in proinflammatory B cells (Revilla et al., 2012). A study of whole-genome DNA methylation in peripheral blood mononuclear cells from influenza vaccine responders and nonresponders across different ages identified potential age-related contributors to vaccine responsiveness (Gensous et al., 2018). Epigenetic and transcriptomic profiles and humoral immune response outcomes among older adults (aged 60 years and older) who were healthy recipients of the influenza vaccine showed that methylation sites are associated with known differentiation-signaling and antigen-presentation pathways (Gensous et al., 2018; Fu et al., 2024). These findings further confirm the role of defective antigen-presenting cells in vaccine efficacy among the elderly. It was found that both mDCs) and pDCs from elderly individuals were significantly impaired in their capacity to secrete TNF-α/IL-6/IL-12 (p40) and in TNF-α/IFN-α production, respectively, in response to TLR1/2, TLR2/6, TLR3, TLR5, and TLR8 engagement by mDCs and TLR7 and TLR9 by pDCs. These defects were strongly associated with poor antibody response and reduced proportion of Influenza virus-specific CD8^+^ T cells (L’Huillier et al., 2020). Stronger CD4^+^ and CD8^+^ T cells are associated with better protection against reinfection; both T cell subsets are reduced in vaccinated elderly (Swain et al., 2012). Furthermore, T cells from influenza-vaccinated elderly individuals exhibit higher expression of PD-1, CD57, and KLRG1, which are associated with reduced functionality and proliferative capacity (Wagar et al., 2011). One factor leading to T cell exhaustion in these individuals is repeated stimulation by persistent CMV infection, which is highly prevalent in older adults. CMV seropositivity is associated with diminished influenza-specific CD8^+^ T cell responses and increased cellular exhaustion. While in young adults, CMV infection can sometimes have a neutral or even beneficial effect on vaccine responses. For example, young CMV-infected adults have demonstrated enhanced antibody responses to influenza vaccines compared with CMV-uninfected counterparts, possibly due to increased innate immune cell function. While influenza vaccines are generally safe for the elderly, their effectiveness is lower in those with CMV-associated immune dysfunction. Latent CMV infection decreases naive T-cells and increases effector CD8^+^ T-cells, often resulting in poorer vaccine-specific antibody responses among elderly individuals (Merani et al., 2017; Frasca et al., 2015). Conversely, B cells mount a complex response that includes rapid IgM secretion and infiltration of the lungs to limit early viral dissemination. However, influenza vaccination leads to lower IgM levels, decreased IAV-specific IgG, and a shorter B cell activation window in the elderly. Moreover, a hemagglutination-inhibition (HAI) antibody titer above 1:40 is regarded as providing 50% protection against influenza in older adults. However, older individuals generally produce lower peak HAI titers following vaccination than younger individuals, and these titers usually decline more quickly. Although influenza-vaccination significantly protects against influnza virus infection among older adults compared to non-vaccinated, however, these age-related immune dysfunction during influenza vaccination leads to poor vaccine response, shorter protection, increased seasonal influenza infection, and hospitalization rates among the elderly.

## Aging and COVID-19 vaccination

The SARS-CoV-2 virus causes coronavirus disease 2019 (COVID-19), an acute respiratory illness that can range from no symptoms to severe pneumonia and respiratory failure. Throughout the pandemic, there has been a widespread increase in hospitalization and mortality rates among the elderly population (Fericean et al., 2023). Most of the COVID-19-associated hospitalizations and deaths were among older adults (Shahid et al., 2020). SARS-CoV-2 vaccination led to a significant reduction in the death rate among elderly individuals, compared with unvaccinated individuals. For example, monocyte analysis showed that older adults with COVID-19 had lower activation, reduced antigen-presenting ability, and increased IP-10 levels. Likewise, aging leads to decreased dendritic cell activation and a lesser capacity for antigen cross-presentation in the early phase of COVID-19 (Cham et al., 2021; Bajaj et al., 2020; de Souza Xavier Costa et al., 2025). In contrast, older adults show increased NK cell activation, cytokine production, and IL-2 secretion (Fionda et al., 2022). Furthermore, advanced age was associated with reduced CD8 T cell activation and earlier exhaustion. Older COVID-19 patients show a higher proportion of effector memory and central memory CD8 T cells compared to younger individuals. Some studies indicate that vaccinated individuals over 65 years of age have higher PD-1 and TIGIT expression levels on spike-specific T cells, which correlate with lower CD8^+^ T cell function and reduced antibody production during vaccination (Rha and Shin, 2021; Westmeier et al., 2020; Murdaca et al., 2023). Moreover, CMV infection has been shown to accelerate the accumulation of terminally differentiated CD8^+^ effector memory cells with limited proliferative capacity. CMV-seropositive elderly individuals often show reduced T-cell interferon-gamma production and IgG antibody levels in response to vaccines. However, studies of the ChAdOx1 nCoV-19 (SARS-CoV-2) vaccine in younger adults showed that CMV-associated terminal differentiation did not significantly reduce overall antibody or cellular responses (Sharpe et al., 2022). Some multicentre longitudinal studies found that Despite these age-related defects, mRNA COVID-19 vaccines generate particularly strong immune responses in the elderly compared to other vaccine platforms, despite the overall age-related reduction. Studies show that elderly individuals develop high antibody titers to mRNA vaccines, often exceeding those seen after natural infection, with seroconversion rates often exceeding 90%. Additional doses or boosters are highly effective at strengthening antibody responses in older adults, often boosting them to levels comparable to those seen in younger individuals (Chen R. R. et al., 2025; Segato et al., 2024). These observations reflect that vaccination remains clinically beneficial, providing significant protection against severe COVID-19 (80%–90% effectiveness), reduced hospitalization, and mortality among elderly individuals (Fedele et al., 2022; Wei et al., 2022). However, these studies indicated an age-related decline in T-cell responses and neutralizing antibody titers. Some findings reported a tenfold decrease in the neutralizing antibody titers in the older group, and a significant reduction in spike-reactive T-cell responses (Chen R. R. et al., 2025). Additionally, studies of other mRNA vaccines have reported significantly lower antibody levels and T cell responses among older adults, and the frequencies of CD4^+^ and CD8^+^ T cells producing spike-specific IL-2, IFN-γ, and IgG were negatively correlated with age (Chen R. R. et al., 2025; Segato et al., 2024). Moreover, aging leads to a loss of T cell receptor diversity in both CD8 and CD4 cells, as well as to reduced overall T cell function and survival. Further studies on T-cell responses showed that IFN-γ and IL-2 were negatively correlated with age (Segato et al., 2024). Analysis of T cells post-vaccination also revealed increased production of short-lived effector T cells relative to memory precursor cells, resulting in an impaired response of T follicular helper cells to vaccination (Bettini and Locci, 2021). Furthermore, B cell numbers remain more consistent with age, but reduced expression of select proteins in older adults results in fewer functional antibodies (Kometani et al., 2024). Furthermore, studies show a 2.5–4.6-fold reduction in neutralizing antibody (nAb) titers 6 months after XBB-containing booster doses. Older age and higher frailty levels are independently associated with lower antibody titers at 6 months, regardless of prior infection history. Individuals aged 90 and older may experience more rapid antibody decay compared to those aged 65–80, despite occasionally having high initial responses (Chen R. R. et al., 2025). Despite these age-related defects, vaccination provides substantial protection and significantly lowers mortality rates in older adults, resulting in a significant reduction in hospitalization and death.

## Aging and pneumococcal vaccination

*Streptococcus* pneumoniae is a Gram-positive, lancet-shaped diplococcus that causes community-acquired pneumonia. Pneumococcal infections are the leading cause of upper and lower respiratory tract infections and a major contributor to morbidity and mortality (Kadioglu et al., 2002). With age, the incidence of pneumococcal pneumonia increases with the number of comorbidities (Cheong and Song, 2024). To date, two pneumococcal vaccines are available for adults aged 60 years and older: the 13-valent pneumococcal conjugate vaccine (PCV13) and the 23-valent pneumococcal polysaccharide vaccine (PPV23). While both vaccines induce an antibody response, PCV13 induces a T cell–dependent immune response that, in turn, drives B cell memory, whereas PPV23 induces antibodies via a T cell–independent mechanism (Lewnard et al., 2022). The distinction between PCV13 (13-valent pneumococcal conjugate vaccine) and PPSV23 (23-valent pneumococcal polysaccharide vaccine) hinges on their immunological mechanisms and durability, especially in older adults (Serra-Prat et al., 2024; Latifi-Navid et al., 2018). PCV13 is a conjugate vaccine that induces a T-cell-dependent immune response, generating long-lived memory B cells and providing more durable protection. PCV13 links polysaccharide antigens to a carrier protein, allowing the immune system to create T-cell-dependent memory, which is essential for lasting immunity. In contrast, PPSV23 acts via a T-cell-independent mechanism, leading to more variable and often waning effectiveness over time, particularly in older adults. PPSV23 is a pure polysaccharide vaccine, resulting in a T-cell-independent response that generally does not create immunological memory (Serra-Prat et al., 2024; Latifi-Navid et al., 2018). Evidence consistently shows PCV13 offers superior protection against vaccine-type pneumonia and IPD compared to PPSV23 in older adults. Studies, including the CAPiTA trial, indicate that PCV13 is more effective (41%–71%) against VT-pneumococcal pneumonia than PPSV23 (2%–6%) in older adults. PCV13 has shown approximately 10% effectiveness in reducing all-cause pneumonia, which is rarely observed with PPSV23. PCV13 tends to provide more durable protection over a 5-year period than PPSV23, which may show reduced effectiveness after the first year. (Le et al., 2024; Theilacker et al., 2022). Despite the PCV13 vaccine’s well-established immunogenicity, older adults exhibit a significantly reduced vaccine response compared with younger individuals (Marra and Vadlamudi, 2019). The mechanism by which older adults respond less effectively to pneumococcal vaccines is widely attributed to immunosenescence and inflammaging. Compared with younger adults, pneumococcal immunization in older adults leads to reduced B- and T-cell activation and function, impaired IgG antibody function, decreased complement components, and fewer neutrophils, resulting in reduced opsonization (Park and Nahm, 2011; Wang B. et al., 2025; Lee and Linterman, 2022). Aging reduces the amount of IgG and the quality of IgG specific to neutralizing pneumococcal infection. Elderly individuals may exhibit lower opsonophagocytic activity compared to younger adults. Although the 23-valent vaccine works well in young adults, its effectiveness decreases in elderly adults. This reduced effectiveness may result from age-related changes in the antibody repertoire and a decline in IgM production, linked to shifts in B cell subpopulations associated with aging (Serra-Prat et al., 2024; Berical et al., 2016). There is a notable reduction in IgM memory B cells (CD27+IgM+) in the elderly (Visser et al., 2025; Shi et al., 2005). While younger adults respond with an increase in these cells, older adults often show a predominantly switched memory B-cell response (CD27+IgM-) (Shi et al., 2005), which may be less effective against encapsulated bacteria. This might explain why the PPSV23 does not produce long-term immune memory, and antibody levels can return to baseline within 3–5 years in the vaccinated elderly (Serra-Prat et al., 2024; Berical et al., 2016). Moreover, older adults exhibit impaired Tfh cell numbers and potentially distinct patterns of T-cell exhaustion compared with younger adults. Studies have shown a trend toward an increased proportion of PD-1 CD4^+^ and CD8^+^ after primary and booster pneumococcal vaccinations among older adults (Park and Nahm, 2011; Wang B. et al., 2025; Lee and Linterman, 2022). Thus, leading to increased susceptibility to pneumococcal infection, greater disease severity, and higher mortality among the elderly. Given the increasing global prevalence of pneumonia-related hospitalizations, vaccines with broader coverage and longer-lasting protection are needed.

## Strategic approaches to enhance vaccine response in the elderly

Given the rising global population of older adults and the emergence and re-emergence of infectious agents, developing strategies to improve vaccine efficacy in this population should be a global health priority. Unarguably, immunosenescence and inflammaging synergistically impair vaccine responses in older adults by reducing the function of the innate and adaptive immune cells. The use of systems biology approaches, high-throughput multi-omics technologies, and computational modeling will be essential for uncovering immunological and molecular mechanisms of aging. In earlier sections, we explored various pathways related to immunosenescence and inflammaging, and targeting these pathways could improve vaccine immunogenicity in the elderly.

Some approaches with immediate applicability to improve vaccine response include developing broader vaccines and adjuvants that target surface proteins or noncapsular antigens, thereby improving the effectiveness of vaccine-induced antibodies in older adults. Adjuvants, or their combination with immunotherapeutic agents, are vital for enhancing vaccine responses in the elderly. For instance, AS03 significantly enhances the magnitude, persistence, and clonal breadth of memory B-cell responses, as seen in COVID-19 and influenza vaccines. AS04 enhances antigen presentation and broadens antibody responses, used in HPV and Hepatitis B vaccines. CpG/TLR9 agonist stimulates Th1 responses and is effective at promoting the expansion of IgG clones. Moreover, increasing the antigen load helps overcome the age-related reduction in naïve T-cell activation and increased activation thresholds for B cells. High-dose and adjuvanted (aIIV3) vaccines are recommended for older adults to overcome immunosenescence by inducing a more robust response, though they still operate within the constraints of established imprinting. Future vaccine strategies may need to account for this lifetime history of exposures to better target conserved, subdominant head or stem epitopes, rather than relying on boosting the heavily imprinted memory B-cell responses. Furthermore, given that gut microbiota contributes to inflammaging and mitigates damage, a mucosal vaccine with an appropriate adjuvant would be an appealing option. Development of newer technologies, such as mRNA vaccines or mRNA boosters after vector-based primary vaccination, can offer improved immunogenicity compared with conventional inactivated vaccines. Although it is still under investigation, intranasal delivery shows promise in triggering local IgA mucosal immunity, which is crucial for respiratory viruses.

Other emerging and future approaches include telomerase, a key ribonucleoprotein enzyme that maintains chromosome length and helps prevent immunosenescence. Aging is associated with suppressed telomerase activity. Therefore, using pharmacological agents such as TA-65, Androgen therapy, and small-molecule activators to boost telomerase expression could slow down immunosenescence. Another potential approach to reducing general cellular aging involves senolytic therapy, which combats aging by targeting and eliminating senescent cells. Notably, senescent cells can accumulate, cause inflammation, and accelerate age-related immune dysfunction. Early clinical trials showing promise report that senolytic drugs help in reversing cellular senescence. Moreover, the use of mTOR inhibitors, such as rapamycin and metformin, can improve T- and B-cell function during vaccination. Targeting specific cells could also help restore vaccine response among older adults. An age-related increase in the accumulation of age-associated B cells (ABCs) impairs vaccine responses and contributes to inflammaging. B-cell therapy targeting the depletion of ABCs and senescent B cells can potentially rejuvenate the B-cell response and improve vaccine responses. Therapies aimed at clearing ABCs can reduce the risk of developing inflammaging.

**FIGURE 3:**
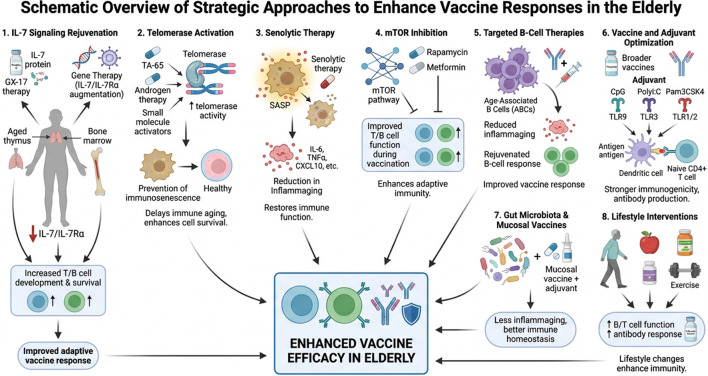
Schematic overview of strategic approaches to enhance vaccine response in the elderly. Overview of potential approaches, such as IL-7 therapy, senolytic therapy, increasing telomerase activity, mTOR inhibitors, B cell therapy, development of broader vaccines and new adjuvants, and positive lifestyle changes, to enhance immune function among the elderly.

Implementing practical, supportive strategies such as regular exercise and lifestyle modifications can help improve vaccine responsiveness in older adults. Regular exercise reduces inflammaging and improves B- and T-cell numbers and function. Regular exercise is strongly associated with better antibody production in response to influenza vaccination. Therefore, positive lifestyle changes, such as a healthy diet, dietary supplements, and increased physical activity, naturally boost telomerase activity, restore B cells and humoral immunity, and lead to better immune response. Figure 3 provides a summarized overview of potential strategies to improve vaccine response in the elderly.

## Conclusion

Vaccination against major respiratory pathogens (RSV, influenza, COVID-19, *S. pneumoniae*) in elderly individuals has been shown to provide significant clinical benefit in preventing severe disease, hospitalization, and mortality. Vaccination among older adults boosts protection by 80%–90%, reduces severe RSV outcomes by over 90%, and significantly lowers mortality rates. A significant reduction in hospitalizations for influenza and the risk of dying from flu-related complications. SARS-CoV-2 vaccination among elderly individuals has significantly reduced hospitalizations, risk of COVID-19 intensive care unit admission and deaths. Pneumococcal vaccination has been associated with a 53% reduction in in-hospital mortality among elderly patients with pneumonia. However, immunosenescence and inflammaging are the most critical age-related factors that contribute to impaired vaccine immunogenicity and efficacy. Effective vaccination is vital for the health of aging populations. Therefore, developing strategic approaches to boost vaccine responses and restore immune function in the elderly is crucial. Comprehensive approaches, including IL-7 therapy, senolytic drugs, increasing telomerase activity, mTOR inhibitors, B cell therapy, development of broader vaccines and new adjuvants, and positive lifestyle changes, may help manage age-related immune decline and promote healthy aging. Successfully applying or combining these strategies will undoubtedly slow immunosenescence, reduce inflammaging, improve vaccine effectiveness and efficacy, reduce morbidity and mortality, and increase overall quality of life for the aging population.
